# Perceived barriers for treatment of chronic heart failure in general practice; are they affecting performance?

**DOI:** 10.1186/1471-2296-6-19

**Published:** 2005-05-03

**Authors:** Willeke N Kasje, Petra Denig, Pieter A de Graeff, Flora M Haaijer-Ruskamp

**Affiliations:** 1 Department of Clinical Pharmacology, University Medical Center Groningen, A. Deusinglaan 1, 9713 AV Groningen, The Netherlands; 2 Department of Internal Medicine, University Medical Center Groningen, The Netherlands

## Abstract

**Background:**

The aim of this study is to determine to what extent barriers perceived by general practitioners (GPs) for prescribing angiotensin-converting enzyme inhibitors (ACE-I) in chronic heart failure (CHF) patients are related to underuse and underdosing of these drugs in actual practice.

**Methods:**

Barriers were assessed with a semi-structured questionnaire. Prescribing data were extracted from GPs' computerised medical records for a random sample of their CHF patients. Relations between barriers and prescribing behaviour were assessed by means of Spearman rank correlation and multivariate regression modelling.

**Results:**

GPs prescribed ACE-I to 45% of their patients and had previously initiated such treatment in an additional 3.5%, in an average standardised dose of 13.5 mg. They perceived a median of  four barriers in prescribing ACE-I or optimising ACE-I dose. Many GPs found it difficult to change treatment initiated by a cardiologist. Furthermore, initiating ACE-I in patients already using a diuretic or stable on their current medication was perceived as barrier. Titrating the ACE-I dose was seen as difficult by more than half of the GPs. No significant relationships could be found between the barriers perceived and actual ACE-I prescribing. Regarding ACE-I dosing, the few GPs who did not agree that the ACE-I should be as high as possible prescribed higher ACE-I doses.

**Conclusion:**

Variation between GPs in prescribing ACE-I for CHF cannot be explained by differences in the barriers they perceive. Tailor-made interventions targeting only those doctors that perceive a specific barrier will therefore not be an efficient approach to improve quality of care.

## Background

Despite several landmark studies showing that appropriate treatment of chronic heart failure (CHF) can improve morbidity and mortality, management in general practice is still not optimal. Persisting major problems are underuse and underdosing of angiotensin-converting enzyme inhibitors (ACE-I) [1].

General practitioners (GPs) perceive problems that may explain why they do not treat their CHF patients optimally [2-5]. These problems can be classified as internal or external barriers. Internal barriers include lack of knowledge, e.g. not knowing the target dose of ACE-I or lack of awareness of new recommendations, as well as certain attitudes, such as lack of confidence, doubts about benefits for very old patients, fear for adverse effects or reluctance to change the treatment when a patient is stable. External barriers may be related to organisational factors, including difficulties at the primary-secondary care interface.

It is often suggested that intervention programs for improving performance need to be targeted at perceived barriers [6,7]. However, several tailored interventions addressing identified barriers did not change professional performance [8,9]. It might be that not all barriers identified are as relevant for not achieving optimal management. In the case of CHF management, lack of knowledge appears not to be very pertinent [10,11]. Furthermore, doctors may not be fully aware of the factors influencing their performance, because self-insight in treatment decisions is limited [12]. To our knowledge, no study has tried to assess the relationship between the GPs' self-reported problems with specific treatment recommendations and their actual prescribing for heart failure. Better understanding of this relation may help to indicate areas in which an intervention could be most beneficial.

The aim of our study is to determine to what extent barriers that GPs perceive for prescribing ACE-I in CHF patients are related to their actual prescribing.

## Methods

### Study population and setting

**Figure 1:**
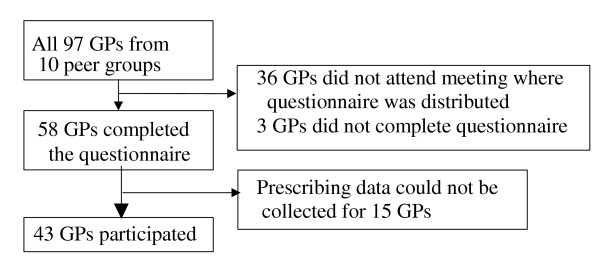
General practitioners (GPs) and patients in study

This study was part of the baseline of a larger study conducted from September 2001 to May 2002 in the north of the Netherlands, evaluating two audit programs for peer review groups focussing on the treatment of CHF and treatment of hypertension in diabetic patients. In the Netherlands, nearly all GPs participate in such peer review groups. Of the 27 peer review groups in our region, 21 participated in the larger study. A total of ten peer review groups consisting of 97 GPs were randomised to the chronic heart failure program, and therefore eligible for this study (Figure 1).

Prescribing data were extracted from the GPs' computerised medical records. In most practices, GPs have personal lists of patients. Data were collected for a random sample of 10 CHF patients per practice using computer generated random numbers. The estimated prevalence of CHF lies between 15–30 patients for an average patient list in the Netherlands of 2400 patients per GP [13]. All patients with a diagnostic code for CHF or the text 'heart failure', 'cardiac asthma', 'cardiac decompensation' or 'left ventricular dysfunction' in their medical records were selected from the medical records as possible CHF patients. GPs were asked to verify the diagnosis. Because of the larger study, CHF patients with a co-morbidity of diabetes type 2 were excluded.

### Perceived barriers

GPs present at the audit meeting of their peer review group were asked to complete a semi-structured questionnaire on their perceived problems with the recommended treatment for CHF. This questionnaire was developed with statements of possible internal and external barriers towards prescribing ACE-I in patients with heart failure as identified from the literature [2-5]. The literature-based barriers included three general beliefs supporting the recommended treatment. Disagreement with these was considered an internal barrier. Six specific beliefs and attitudes opposing the general recommendations were presented, each also representing a possible internal barrier. Furthermore, two external barriers were included which were related to the sharing of responsibilities between primary and secondary care (Table 1). An open-ended question was added to identify any other barriers the GPs perceived with implementing evidence-based recommendations for CHF treatment. These self-reported barriers were categorised in nine themes by the first two authors. A content analysis based on an inductive approach was conducted. First, the two authors independently identified the main issues described by the GPs. This resulted in thirteen issues that after comparison and discussion were reduced to nine separate themes. Next, both researchers independently classified all reported barriers to one of the nine themes. Discrepancies were discussed until agreement was reached.

**Table 1:** Perceived internal and external barriers for prescribing ACE-I for CHF, divided in literature-based and self-reported barriers (N = number of GPs reporting barrier)

	Literature-based barriers	N	Self-reported barriers	N
Internal	*Do not agree with*: I believe that the standard therapy for new CHF patients should be an ACE-I, irrespective of the severity of the disease	1		
	I believe that the standard therapy for known CHF patients should be an ACE-I, irrespective of the severity of the disease	2		
	I believe that ACE-I should be prescribed in as high a dose as possible for CHF patients	2		
	
	*Agree with*: I believe one should be reserved in prescribing ACE-I to CHF patients, because of the risk of renal insufficiency	11	Starting, checking, and titrating ACE-I dose is difficult	3
	I believe one should be reserved in prescribing ACE-I to CHF patients, because of the risk of hypotension	12	Fears about adverse effects of ACE-I	8
	I find initiating ACE-I difficult in CHF patients already using a diuretic	18		
	I find it difficult to frequently titrate the ACE-I dose in CHF patients	25		
	I believe that CHF patients who are stable on their current medication, should not be put on an ACE-I	18	Not wanting to change treatment when patients are stable	4
	I believe it is not useful to prescribe ACE-I to very old CHF patients	10	Doubts about usefulness of ACE-I, especially in elderly patients	3
			Difficulties with treating complex cases (comorbidity/polyfarmacy)	3

External			Problems with patient compliance or motivation	5
	I believe that a cardiologist should initiate ACE-I therapy in CHF patients	3	Problems in interacting with specialist care	9
	I find it hard to change treatment initiated by a cardiologist	33		
			Time constraints	1
			Difficulties with screening for undertreated heart failure patients	4

### Actual treatment

Actual treatment data were extracted from computerised medical records by third and fourth year medical students trained to copy all relevant medical and prescription data on structured forms. When two GPs shared their list of patients, prescribing decisions were assigned to both GPs reflecting their joint prescribing policy. Data collected for the patients included age, gender, date of CHF onset, specialist referrals and current medication. For each GP, the percentage of patients currently or previously treated with an ACE-I was calculated as outcome variable, as well as the average dosing of the ACE-I currently prescribed. The ACE-I dosages were first converted to standardised dosages according to target daily doses for heart failure as recommended in the Dutch desk reference book [14]. This method of standardisation, which has been used before, uses 20 mg of enalapril as reference dose [15]. Based on the conversion, enalapril 20 mg equivalents are captopril 150 mg, ramipril 10 mg, quinapril 20 mg, lisinopril 20 mg, fosinopril 40 mg, and perindopril 4 mg. This conversion is an alternative for the more commonly used defined daily dosage (DDD) method, which can not be applied in this case since the DDDs for ACE-I are based on their use for hypertension.

### Data analysis

Prescribing of ACE-I was aggregated at GP level to assess the relationship between the perceived barriers and overall prescribing behaviour. We checked at patient level with chi-square tests whether ACE-I prescribing differed significantly for different age-groups, gender and comorbidity of the patients. Differences between participating and non-participating GPs were tested with t-tests or chi-square tests.

The number of different barriers perceived was related to the percentage of patients currently or previously being prescribed an ACE-I and to the average standardised ACE-I dose prescribed with Spearman rank correlations (ρ). This non-parametric statistic was used since the number of barriers perceived did not have a normal distribution. Two sub-analyses were conducted to assess whether relations differed for internal versus external barriers, and for literature-based versus self-reported barriers. Next, we looked at the relationship between individual barriers and actual ACE-I prescribing. The data were first explored univariate by means of Mann-Whitney tests. A stepwise multivariate linear regression model was used to assess the relevance of all 11 literature-based and five self-reported barriers for explaining differences in ACE-I prescribing aggregated at GP level. The other self-reported barriers overlapped with literature based barriers and were excluded from this regression analysis. Finally, the complete data analysis was repeated for ACE-I prescribing for a subgroup of patients who had not been referred to a cardiologist in the year prior to data collection. This was decided when it became clear that a substantial number of patients had been referred in the last year, and one might expect that treatment initiated by the specialist confounds the analysis [16].

## Results

Fifty-eight GPs completed the questionnaire, and prescribing behaviour was measured for 43 of them, resulting in an overall response rate of 44% (Figure 1). The GPs participating in this study were mainly male (88%), and on average 47 years old (SD 6.9), which was not significantly different from the non-responding GPs (83% male, 48 years). They had an average practice size of 2485 patients (SD 244) and 60% were single-handed. In comparison, the average Dutch practice size in 2001/2002 was 2430 patients, and 40% of the GPs was single-handed. In thirteen practices, less than ten verified CHF patients could be identified. In another three practices, two GPs shared the responsibility for the same patients. Prescribing behaviour was therefore assessed for 339 patients. In 11% of the cases, the diagnosis was confirmed by a recorded echocardiography. The GPs prescribed an ACE-I to an average of 44.9% (SD 15.9) of their CHF patients in an average standardised dose of 13.5 mg (SD 6.6). Another 3.5% of the patients had been using an ACE-I prior to the study period, including 7 patients that had stopped using ACE-I because of various side effects and 5 who had stopped without a documented reason. Including these patients, the GPs prescribed or had previously prescribed an ACE-I to 48.6% of their patients (SD 17.9). This was not significantly different from GPs who did not attend the meeting (47.3%). ACE-I prescribing was significantly lower for patients over 85 years of age (32.8%). ACE-I prescribing did not significantly differ according to the patients' gender or comorbidity. Angiotensin-II-antagonists were prescribed to 6% of the patients.

All but two GPs considered ACE-I as standard therapy for all CHF patients, and most GPs agreed that ACE-I should be dosed as high as possible (Table 1). The median number of barriers perceived in prescribing ACE-I or optimising ACE-I dosage was four. All 43 GPs perceived at least one barrier; 41 GPs perceived at least one internal barrier, and 37 GPs perceived at least one external barrier (Table 2).

**Table 2:** Number of perceived barriers and average ACE-I prescribing in CHF patients (N = number of GPs)

**Number of barriers**	**N**	**% patients on ACE-I**	standardised ACE-I dose (mg)*
1	1	80.0	13.7
2	9	49.7	13.5
3	7	42.2	10.9
4	11	48.1	15.0
5	7	41.4	15.4
6	4	67.1	13.4
7	2	31.7	11.2
8	1	62.5	6.2
10	1	55.6	9.2
median = 4.0 (SD 1.86)	43	48.6	13.5
median internal barriers = 3.0 (SD 1.64)	41	48.5	13.5
median external barriers = 1.0 (SD 0.88)	37	47.1	12.9

* = based on a conversion using a standardised target dose of 20 mg

### Relationship between number of barriers and ACE-I prescribing

No relationship appeared to exist between the number of barriers and the ACE-I prescribing at GP level (Table 2). No significant correlations were found between the total number of barriers perceived by the GPs and the percentage of patients receiving an ACE-I (ρ = .02) or the average ACE-I dose prescribed (ρ = -.08). Also, no significant correlations were found with the number of internal or external barriers, nor with the number of literature-based or self-reported barriers.

### Literature-based barriers

With regard to initiating an ACE-I, a substantial number of GPs (42%) reported that they were afraid of endangering a stable situation and reluctant to start an ACE-I when a patient already received a diuretic (Table 1). The most important barrier regarding the ACE-I dosing was the difficulty perceived with titrating this dose. A majority of the GPs (77%) found it hard to change treatment initiated by a cardiologist. The univariate analysis showed no significant relationships between the individual barriers and ACE-I prescribing, and the scatter plots also revealed no patterns. Even GPs who believed it is not useful to prescribe ACE-I to very old CHF patients did not have less patients of 85 years or older on these drugs (univariate correlation ρ = .19, p = 0.3). In the stepwise linear regression model using forward selection, none of the 11 literature-based barriers was found to be related to the percentage of patients currently of previously receiving an ACE-I. The model as a whole did not significantly explain the prescribing differences at GP level (R-square = 0.17). Also, a model including only the five internal barriers directly related to ACE-I prescribing, i.e. fear of renal insufficience, fear of hypotension, difficulty in initiating ACE-I in patients on diuretics, not wanting to start ACE-I in stable patients, not wanting to prescribe ACE-I to very old patients, could not predict ACE-I prescribing (R-square = 0.10).

Surprisingly, the two GPs who did not agree that the ACE-I dose should be as high as possible, prescribed higher doses than GPs who did agree with this recommendation. This barrier was significantly associated in the multivariate linear regression model explaining differences in ACE-I dosages (beta 0.42, p = .03). The GPs who agreed that it is not useful to prescribe ACE-I to very old CHF patients were prescribing lower ACE-I doses (beta 0.34, p = .04).

### Self-reported barriers

The most common self-reported barrier concerned the sharing of responsibilities with specialists (Table 1). According to several GPs co-management with a cardiologist made it difficult to change the therapy, and some GPs felt the cardiologist interfered too much. Furthermore, eight GPs mentioned possible adverse effects of ACE-I as a barrier towards prescribing. A few GPs mentioned problems with patient motivation as a barrier. Addition of the self-reported barriers to the multivariate model did not significantly alter any of the findings.

### Analysis of patients not referred to a cardiologist

Cardiologist treatment could have confounded our analysis. Patients not referred to a cardiologist in the year prior to data collection were prescribed an ACE-I less often than the 36% of patients that had been referred (44% versus 61% on ACE-I, t-test = -2.2, p = .03). No significant difference was found regarding ACE-I dosage. Analysis including only the non-referred patients, however, hardly changed our findings. Again no relationship was found between the number of barriers and ACE-I prescribing. In the multivariate model, there were no barriers significantly explaining differences in the percentage of patients currently or previously receiving an ACE-I. One additional factor was found to be associated in the model explaining differences in ACE-I dosages. GPs who believed that CHF patients stable on their current medication should not be put on an ACE-I prescribed higher dosages of ACE-I (beta -0.48, p = .02).

## Discussion

In this study we found remarkably few relationships between perceived barriers and actual prescribing for CHF. The problems that certain GPs acknowledged, such as their reluctance to initiate ACE-I in already treated CHF patients or the difficulties with gradually increasing the ACE-I dose, were not reflected in their prescribing of these drugs. No matter what barrier GPs report, it does not seem to affect their management of CHF patients in general practice.

For some patients, GPs tried to initiate an ACE-I but treatment had been stopped for various reasons. We included these cases in our analysis, thereby focussing on all attempts of a GP to start ACE-I treatment in CHF patients. A third of the patients in our study were seen by a cardiologist in the year prior to data collection, which was found to be related to receiving more ACE-I. However, subgroup analysis including only prescriptions for patients not recently referred to a cardiologist did not show any concealed relationships.

In our study we took the overall prescribing of ACE-I for CHF patients at GP level as primary outcome measure, expecting to find relationships between perceived barriers and the general prescription pattern. Since ACE-I should be started in all CHF patients, this aggregated measure is considered a relevant performance indicator for the CHF treatment [17]. At patient level, however, we did observe a lower prescription rate for patients over 85 years of age. Therefore, we decided to look at the specific association between the barrier for prescribing ACE-I to very old patients and actual ACE-I prescribing in this subgroup. Even on this specific level no significant relationship could be found. Our findings are in line with those from a recent explanatory study on effective management of type 2 diabetes, where no relationship was found between the presence of barriers perceived and the number of recommendations followed by physicians [18].

The representativity of our GP population should be considered. The 43 responding GPs were representative regarding age and gender for the total of 97 GPs enrolled in the larger study, and there were also no differences regarding their prescribing of ACE-I for CHF. In comparison to all Dutch GPs, our study population included a relatively large proportion of single-handed, male GPs that is typical for our region. A previous study showed, however, that such general physician and practice characteristics did not determine ACE-I prescribing for CHF [19]. Therefore, we do not expect that the regional selection limited the analysis of the relationship between perceived barriers and actual prescribing. Another matter of concern is the power of our study to detect relevant associations. We analysed all data on the GP level, since barriers were measured at this GP level and not linked to individual patients. Our sample size of 43 achieved an 80% power to detect moderate correlations of 0.41 in the univariate analyses. In the linear regression model, this sample size achieved an 80% power to detect an R-squared of 0.34 attributed to 11 independent variables or an R-squared of 0.26 attributed to 5 variables. This implies that there may have been weaker associations that we have missed both in the statistical analysis and by inspecting at the univariate scatter plots. We used medical records to measure the GPs' prescribing behaviour for CHF patients. As is the case with more than 90% of Dutch GPs, the GPs in this study prescribe electronically, and these prescriptions are automatically stored in the medical records. Patients were selected as having CHF according to their GP, without independently confirming the diagnosis. This was done since our study sought to relate perceived barriers with current management for those patients who the GP considered as having CHF. The low percentage of diagnoses confirmed by echocardiography reflects reality in Dutch primary care [11,20]. The average number of 33 CHF patients identified per practice was in accordance to prevalence rates found in other general practice registrations in The Netherlands [13]. In thirteen practices, less than 10 verified CHF patients could be identified. This was partly due to exclusion of patients with diabetes co morbidity, and partly due to a relatively young patient population in these practices.

## Conclusion

Interventions to improve quality of care often focus on education and addressing perceived barriers for optimal performance. The findings from our study imply that targeting only those doctors that perceive a specific barrier with a tailor-made programme will not be an efficient approach. Variation in the quality of care between GPs can not be explained by differences in the barriers they perceive. It might even be true that being aware of a barrier stimulates some doctors to be more active in dealing with that barrier.

## Competing interests

The author(s) declare that they have no competing interests.

## Authors' contributions

WK carried out the data collection, analyzed the data and drafted the manuscript. PD participated in the design of the study, participated in the statistical analysis, assisted to draft and revise the manuscript. PG participated in drafting the manuscript. FH-R participated in the study design and helped to draft the manuscript. All authors read and approved the final manuscript.

## Pre-publication history

The pre-publication history for this paper can be accessed here:


